# Specialist physicians’ and management personnel's views on climate change mitigation and adaptation in German healthcare facilities: A nationwide survey on attitudes, implementation, and barriers

**DOI:** 10.1016/j.joclim.2025.100602

**Published:** 2025-11-06

**Authors:** Sophie-Charlotte Sasse, Frederick Schneider, Neal Conway, Claudia Doblinger, Kai Kolpatzik, Christian M Schulz, Adrian A W Baumann, Nikolaus Christian Simon Mezger

**Affiliations:** a Technical University of Munich, TUM School of Medicine and Health, TUM University Hospital Rechts der Isar, Department of Anaesthesiology and Intensive Care, Munich, Germany; b Technical University of Munich, Campus Straubing for Biotechnology and Sustainability, Straubing, Germany; c Stiftung Gesundheit, Hamburg, Germany; d KLUG – Deutsche Allianz Klimawandel und Gesundheit e.V., Berlin, Germany; e Global and Planetary Health working group, Medical Faculty, Martin Luther University, Halle (Saale), Germany; f Institute of Environmental Medicine, Karolinska Institutet, Stockholm, Sweden; g Centre of Excellence for Sustainable Health, Karolinska Institutet, Stockholm, Sweden

**Keywords:** Climate change mitigation, Climate change adaptation, Healthcare leadership, Change management, Germany

## Abstract

**Background:**

Climate change poses major challenges for health systems, making mitigation and adaptation measures in healthcare facilities urgent**.** However, little is known about how this is viewed at a healthcare facility leadership level in Germany.

**Methods:**

In September 2022, a nationwide survey was conducted among a representative subset of specialist physicians and healthcare facility management personnel in Germany. As decision-makers in healthcare, this group was surveyed to assess personal attitudes toward climate change and climate-related actions, and healthcare facility-based implementation of mitigation and adaptation measures, as well as possible barriers.

**Results:**

Most of the 514 respondents expressed confidence in their ability to contribute to climate change mitigation and a sense of responsibility to do so. Participants indicated that several structural barriers prevented them from taking necessary climate change mitigation and adaptation action at their facilities. A lack of specifically allocated staff, funding, and poorly defined implementation strategies were the most frequently mentioned constraints. Additionally, the respondents indicated a number of measures which their respective facilities had thus far failed to introduce, such as facility-based heat action plans, education programs, and the integration of sustainability into quality control.

**Conclusion:**

Despite high awareness and willingness among healthcare decision-makers, climate change mitigation and adaptation measures are poorly implemented in German healthcare facilities. Limited strategies, expertise, staff, and funding may be key barriers. The results highlight the need for stronger governance, funding, and performance metrics to support climate action in German healthcare.

## 1 Introduction

The climate crisis has far-reaching effects on ecological, economic, and social systems including healthcare. It leads to more frequent extreme weather events, growing social inequality, worsening mental health [1], and increased morbidity and mortality rates [2]. At the same time, the healthcare sector is a significant contributor to greenhouse gas emissions, thereby feeding a vicious cycle [3]. During the ongoing challenges to health and the economy posed by global political instability and declining investments in the welfare state, healthcare systems are grappling with escalating energy costs [4]. Combined with a growing shortage of skilled labor and the simultaneous overutilization of medical services, this exposes significant deficits in the capacity and resilience of health systems [5,6]. To safeguard the public health achievements of the past century, substantial transformations within the healthcare sector are required [1].

Globally, nonprofit organizations have played a key role in advancing sustainability in the health sector. A WHO-recognized, ten-part framework for healthcare facilities addresses leadership, waste, food, pharmaceuticals, and procurement, among other areas [7]. In response, hospital associations in Germany are beginning to make climate neutrality a priority and explore viable pathways to achieve it [8–10]. For example, recent proposals include a €31 billion investment program to support sustainability measures such as energy-efficient infrastructure [11]. However, a 2021–2022 survey found that while 71 % of hospitals included climate mitigation in their strategies, only 38 % set energy-saving targets and just 30 % assigned staff to sustainability—highlighting the gap between awareness and action [12]. The outpatient sector—including private practices and medical centers—remains largely under-researched, with limited structural or financial incentives for climate action [13,14].

Climate change adaptation and climate-smart occupational health programs are also critical to ensuring healthcare facilities remain functional during more frequent extreme weather events. However, many facilities lack comprehensive plans, often due to unclear responsibilities, weak coordination, and poor integration within municipal preparedness strategies [15]. Further, little is known on implementation of climate-smart occupational health programs at healthcare facilities.

Given its economic output of €392 billion in 2021, its large workforce, and its high standing in society, the German healthcare sector holds substantial potential for a transformation that could resonate well beyond the sector itself [16,17]. In 2021, the German Medical Congress declared the goal of climate neutrality in the German healthcare sector by 2030, and called on all healthcare stakeholders to actively contribute to this effort. Climate change mitigation and resource conservation, coupled with improved adaptation to changing demands are the prerequisites for sustainable healthcare [18,19]. National and international studies suggest that health professionals are concerned about the health effects of climate change and express a willingness to implement mitigation measures within healthcare. However, most of the studies were limited to individual specialties or employment settings and used convenience-based sampling, which may limit the generalizability of their findings [13,20–23]. Further, the role of leadership in shaping climate-smart cultures within healthcare facilities has long been overlooked, but recent studies highlight it as a key factor in promoting compliance, motivation, and long-term commitment [24,25].

This study surveyed healthcare facility management personnel and specialist physicians across all disciplines and facility types within the German healthcare sector. They serve as key decision-makers in strategic planning, institutional governance, and the allocation of resources — functions essential for the implementation of climate-related measures. The survey aimed to assess personal attitudes toward climate change and climate-related actions, the extent to which mitigation and adaptation measures have been implemented at facility level, and the barriers that hinder their adoption.

## 2 Materials and methods

In September 2022, the authors, in collaboration with Stiftung Gesundheit (SG)—a non-profit foundation that maintains a directory of healthcare providers and conducts research in the health sector— carried out a nationwide, anonymous, voluntary, cross-sectional survey of specialist physicians and management personnel in healthcare facilities.

### 2.1 Questionnaire design

**Fig. 1:**
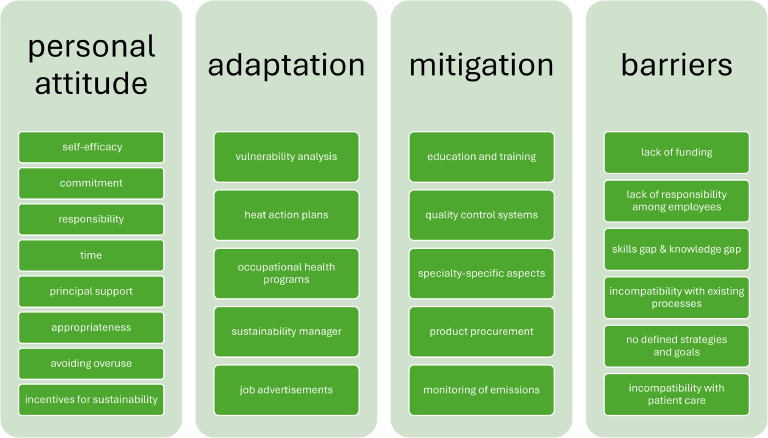
**Overall structure of the questionnaire** (full questionnaire available in Appendix).

An online questionnaire consisting of 24 items was developed to survey healthcare decision-makers. The questions were grouped according to personal attitude toward climate change and related actions, implementation of measures for adaptation and mitigation, and possible barriers to climate change mitigation at healthcare facilities (see Fig. 1 and questionnaire in **supplemental materials**).

The tailored survey instrument was developed by the authors, a multidisciplinary team with expertise in clinical medicine, healthcare systems and policy, innovation management, and planetary health. It was based on findings in change management literature, particularly the concept of "readiness for change" [[26], [27], [28], [29], [30]]. An earlier version of the questionnaire had been designed and piloted in a study on sustainability in anesthesiology at a major German university hospital [23]. For the present study, the instrument was systematically adapted to be applicable across all medical specialties and for healthcare management personnel.

Psychological and structural readiness are considered critical components for change management and were refined and translated to the healthcare setting based on a targeted review of relevant primary sources [26–30]. As recommended in literature, items were selected to adequately reflect the specific research aims: The survey’s personal attitude dimension aimed to capture individual conviction in the success of change initiatives and therefore drew on questions and constructs related to psychological readiness. The barriers dimension was intended to reflect structural and organizational conditions that influence change processes and as such incorporated elements of structural readiness. In addition, items related to facility-based climate change mitigation and adaptation measures were derived from key thematic priorities identified by the Lancet Countdown on Climate Change and Health and the resolutions of the 2021 German Medical Congress [1,18].

While no validated healthcare facility-specific survey instrument was available for the purpose of this study, the questionnaire underwent multiple rounds of expert review to ensure content validity and internal consistency. The items were reviewed, discussed, and refined by the author team and external experts to ensure clarity, relevance, and coherence.

Fourteen items were presented as five-point Likert scales (“fully applies” to “does not apply at all”). Another eight items included "yes," "no," and "I don't know"/"neutral". Two items contained content-specifying response options if at least one "yes" was indicated in questions 2.1 to 2.7.

### 2.2 Sampling frame and study population

A total of 21,079 specialist physicians and management personnel in healthcare facilities were invited to the survey.

The sampling frame was provided by SG’s directory of healthcare providers (Strukturverzeichnis der Versorgung, www.stiftung-gesundheit.de/strukturverzeichnis/), a database on approximately 800,000 healthcare providers and on 400,000 healthcare facilities nationwide). Maintained and updated for over 30 years, it is one of the most comprehensive databases for identifying physicians and other healthcare providers in Germany, used by nearly all statutory and private health insurers in Germany, and containing metadata including specialization. While the directory captures the broader healthcare workforce, it most robustly maps specialist physicians and leadership personnel. As such, this subgroup listed in the directory formed our sampling frame and reflects relevant healthcare decision-makers.

From this directory, a randomized and stratified subset of 15,000 specialist physicians was drawn. All 6,079 medical and administrative managers and directors, as well as managing directors in hospitals and ambulatory medical care centers (“MVZ”) listed in the directory were also invited to participate in the survey. The invited specialist physicians included both inpatient and outpatient physicians. Inpatient physicians included department heads (“Chefärzt:innen”), senior consultants (“Oberärzt:innen”), and specialists (“Fachärzt:innen”), while outpatient physicians in the specialist physician group were practice owners (“Praxisinhabende/-teilhabende”). The composition of the specialist physician group was stratified by gender (self-reported, binary: male, female), age (self-reported, numeric), specialty, contract type, inpatient physicians and physicians in outpatient practice, healthcare setting (primary care/specialty care), and geographic catchment area (all self-reported) to mirror the sampling frame.

### 2.3 Survey

The personalized invitation to the online questionnaire was sent by SG via email. The survey was conducted over a 2-week period (Sept. 13–27, 2022), including a single reminder after one week, and did not offer any incentive for participation. All (fully or partially) answered questionnaires were included in the analysis. Informed consent was obtained from all study participants who consented to anonymized collection and encrypted storage of data by SG before the start of the survey. A positive ethics vote was obtained from the Technical University of Munich (No. 632/20 S). SG was able to provide anonymized sociodemographic data on the 21,079 invited individuals thanks to its database, so that further collection of such data via the questionnaire was deemed unnecessary and therefore not carried out.

### 2.4 Statistical analysis

As respondents could choose to skip individual questions, the response data does not include input from each respondent on every item. For further analysis, the five options of the Likert scale were grouped into affirmative, neutral and non-affirmative responses. In addition to unweighted descriptive statistics, exploratory subgroup analyses were performed with Pearson's Chi² tests and Mann-Whitney-U tests for specialty, facility, and position. R version 4.2.1 and SPSS Statistics version 28 were used for analysis.

## 3 Results

### 3.1 Sample

433 specialist physicians and 81 healthcare facility management personnel responded to the survey and were included in the analysis, corresponding to a response rate of 2.9 % of the invited specialist physicians and 1.3 % of the invited managers. Compared with the study population, respondents were more likely to be female (42.2 % vs. 34.4 %) and slightly younger (median 58 vs. 61 years), more likely to be outpatient practice owners (88.2 % vs. 71.4 %) or managers at a hospital (77.8 % vs. 63.4 %). The distribution by specialty and region of respondents can be derived from **Supplement Table 1**.

**Supplement Table 1**. Demographics of random study population invited and sample of respondents.

### 3.2 Personal views

**Fig. 2:**
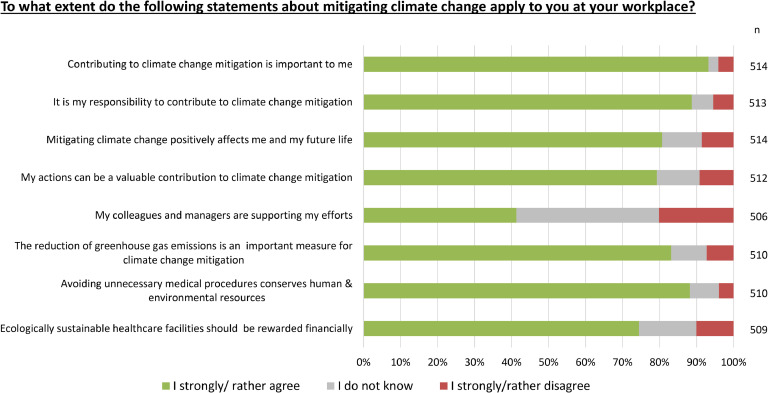
**Personal views on climate action**. Stacked bar chart reflecting the distribution of affirmative (strongly/rather agree), neutral (do not know) and negative (rather/strongly disagree) responses as percentages of the total number of responses (n) to each question.

Among all respondents, 93.2 % believed it was important to contribute to mitigating climate change, and 88.7 % also felt a personal responsibility to do so. The vast majority (79.3 %) agreed that taking climate action at their own healthcare facility could make a valuable contribution and that reducing overall emissions in the healthcare sector was an important measure (83.1 %). Among respondents, 41.3 % stated that they felt supported by colleagues and supervisors in their efforts to address climate change mitigation in the workplace. Nearly all respondents (88.2 %) believed that avoiding unnecessary medical treatments would preserve both human and environmental resources and almost three-quarters of respondents (74.5 %) believed that ecologically sustainable healthcare facilities should be rewarded financially (see Fig. 2).

### 3.3 Climate change adaptation measures

**Fig. 3:**
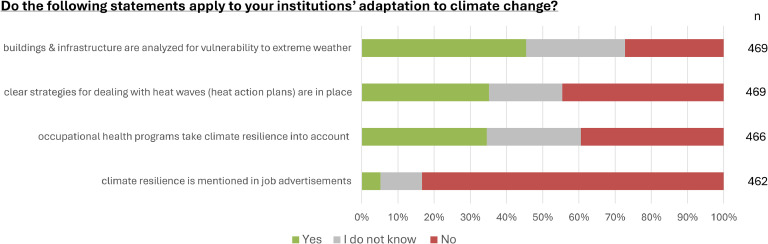
**Adaptation to climate change in the healthcare facility**. Stacked bar chart reflecting the distribution of affirmative (strongly/rather agree), neutral (do not know) and negative (rather/strongly disagree) responses as percentages of the total number of responses (n) to each question.

Less than half of respondents (45.4 %) indicated that their facility's infrastructure had been subjected to analysis to identify vulnerabilities related to heat waves, storms, or floods. A little more than a third of respondents stated that heat action plans were in place in their workplace (35.2 %, see Fig. 3). Such facility-based heat action plans were less commonly reported by respondents working in hospitals (23.6 %) and medical care centers (33.3 %) than by respondents working in outpatient practice (39.6 %). 34.5 % stated that occupational health programs at their facilities took climate resilience into account. Finally, 5.2 % of respondents indicated that climate resilience was mentioned in job advertisements at their facility.

### 3.4 Climate change mitigation and corporate management

**Fig. 4:**
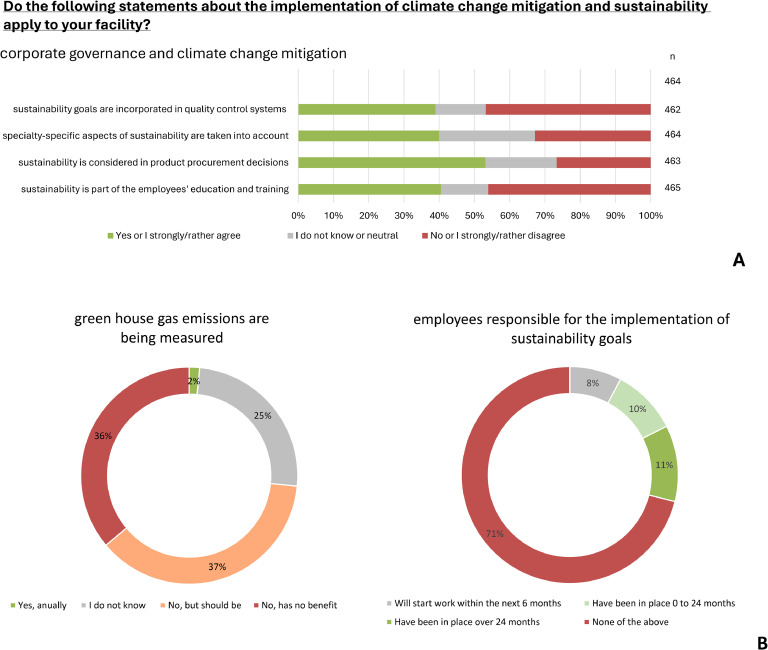
**A and B: Corporate management, climate change mitigation measures and further questions on climate change mitigation and adaptation in the healthcare facility**. Stacked bar chart and pie chart reflecting the distribution of affirmative (strongly/rather agree), neutral (do not know) and negative (rather/strongly disagree) responses as percentages of the total number of responses (n) to each question.

Less than half of respondents stated climate change mitigation was part of training and education concepts for employees (40.5 %), or that climate change mitigation was relevant to quality control at their healthcare facility(39.0 %). In terms of implementing climate change mitigation measures, 11.4 % of respondents stated that their facility had been employing staff specifically responsible for that area for over two years before the time of the survey, whereas more than two-thirds of respondents indicated that their facilities employed no such staff at all (71.1 %) (see Fig. 4**A**.

### 3.5 Climate change mitigation measures at the facility

Six respondents (1.6 %) stated that greenhouse gas emissions were being monitored at their facility. Management staff in particular did not know whether emissions were being tracked (44.6 % vs. 21.7 % of physicians), while the benefit of such assessments was called into question by more than a third of respondents (36.1 %), especially in the outpatient sector (43.3 % in practices/ medical care centers vs. 7.3 % in hospitals). Two fifths of all respondents confirmed that aspects of climate change mitigation and adaptation specific to their departments’ medical specialty were being taken into consideration at their facility (40.0 %) with no significant difference in response tendencies among the specialties. More than half of the respondents (53.1 %) indicated that sustainability was considered in product procurement at their facility. However, it was found that managers were less informed about the topic than specialist physicians (33.8 % vs. 17.6 %) (see Fig. 4**B**).

### 3.6 Barriers to climate change mitigation in respondents’ healthcare facilities

**Fig. 5:**
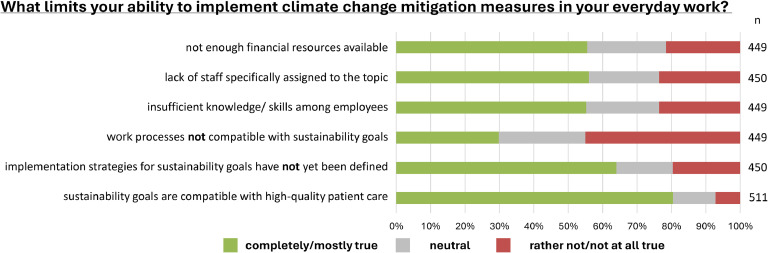
**Barriers to climate change mitigation in the healthcare facility**. Stacked bar chart reflecting the distribution of affirmative (strongly/rather agree), neutral (do not know) and negative (rather/strongly disagree) responses as percentages of the total number of responses (n) to each question.

Respondents highlighted insufficient funding (55.5 %), and insufficient knowledge and skills among staff (55.2 %) as obstacles to reaching sustainability goals at their facility, as well as a lack of staff specifically assigned to their implementation (56.0 %). Two-thirds of respondents also stated that sustainability goals had not yet been defined (64.0 %). In fact, 16.4 % replied that they did not know whether such a strategy existed at all. Almost a third (29.8 %) of respondents indicated that their current working practices were not compatible with climate change mitigation. Meanwhile, 80.4 % of respondents felt that climate change mitigation was generally compatible with high-quality patient care. (Fig. 5) Management staff at healthcare facilities were more likely to report a lack of financial resources for climate change mitigation than physicians (69.4 % vs. 52.8 %). Specialist physicians and managers working in hospital settings criticized the absence of specifically allocated staff at their facility more frequently than their counterparts in outpatient practice and outpatient clinics (70.7 % and 75.5 % vs. 50.8 % and 50 %).

## 4 Discussion

The results of our survey show that many decision-makers in the German healthcare sector recognize the importance of climate change mitigation and adaptation and demonstrate a strong willingness to engage in the implementation of corresponding measures. The vast majority of respondents did not perceive a conflict of interest between delivering high-quality healthcare and implementing climate change mitigation measures at their place of work. Likewise, most respondents endorsed financial incentives that encourage environmentally sustainable practices in healthcare facilities. However, the participants perceived significant shortcomings in the actual implementation of climate change mitigation and adaptation measures.

Less than half of respondents (45.4 %) indicated that their healthcare facility had conducted vulnerability assessments related to extreme weather events, and only 35.2 % of respondents stated that their healthcare facility clearly defined strategies regarding extreme temperatures. This comes in spite of the evidence that heat waves not only put staff and patients at risk [31] but can also result in considerable additional workload and even mass casualty events [32,33]. This aligns with other findings from Germany showing that, though constrained by financial limitations, short-term heat mitigation measures like cooling rooms for staff or hydration support for patients are slowly beginning to be more commonly established [34]. A key barrier appears to be that adaptation measures in healthcare remain voluntary and are supported by limited funding, while the recently enacted German Climate Change Adaptation Act does not explicitly address healthcare facilities [[35], [36], [37]]. Further, studies investigating the effectiveness of healthcare facility-based heat adaptation measures are lacking [38,39]. In addition to insufficient infrastructural adaptation, occupational health programs that support climate resilience remain underutilized tools, reported by a mere 34.5 % of respondents. This seems a missed opportunity, particularly given the dual benefit of such measures for both employee wellbeing and climate change mitigation. MacNeill et al.'s planetary healthcare framework calls for reducing health service demand through improved public health measures addressing social determinants of health—such as promoting active transport, healthier diets, and mental wellbeing [40]. Embedding such measures in German healthcare settings could significantly bolster climate resilience [41,42].

Under 40 % of participants reported that their facility included sustainability goals in its quality control systems, and a lack of educational measures even though specific knowledge and training may be key facilitators of individuals’ commitment to climate change mitigation [23,43]. This contrasted with international examples showing that embedding sustainability into quality improvement and professional development can reduce emissions and cut costs [44]. Respondents largely agreed that avoiding unnecessary treatments helps conserve both human and ecological resources—highlighting alignment between high-quality care and sustainability goals. Other findings have shown that climate change mitigation is often perceived secondary to providing the best possible medical care for individual patients, reflecting a broader debate on overuse and the compatibility of patient care and climate action [45,46]. Initiatives aiming to reduce overtreatment are inherently linked to sustainability but are rarely evaluated through that lens [47,48]. Future studies might therefore examine their impact not only on clinical outcomes but also on climate change mitigation in both outpatient and inpatient settings. Embedding these principles more widely in healthcare could find broad support and may help shift a culture of reactive climate awareness to one of proactive planetary stewardship [40].

Only one-tenth of respondents reported their facility had assigned specific employees to the implementation of climate change adaptation and mitigation, and only a few stated that their facility planned to do so within 6 months of this survey. This gap reflects broader findings from German studies, which point to a significant shortage of facility-based sustainability personnel and weak implementation support [49]. Hospital-level engagement in climate action appears to vary and is influenced by how leadership prioritizes, communicates, and structures sustainability efforts [24]. While understanding a facility’s greenhouse gas emissions may be a key step toward effective transformation [15], only 1.6 % of respondents indicated that such emissions are currently being monitored at their institution. In this context, the recently enacted Energy Efficiency Act may drive progress, as it will require hospitals to track energy use and emissions and submit savings plans beginning in 2025. Supporting tools—such as a recently developed, open-access greenhouse gas emissions calculator tailored to German hospitals—could further assist in setting priorities and monitoring mitigation efforts [50].

In line with recent surveys [9,10,13], more than half of the respondents believed insufficient funding was available for climate change mitigation at their workplace. Although some facilities are already taking steps toward climate change mitigation without dedicated funding, financial barriers remain a major obstacle to implementing sustainability measures in healthcare. Most respondents supported linking hospital reimbursement to sustainability criteria, suggesting that incentive-based models could promote financially viable climate action [8]. Certification frameworks that audit environmental criteria and provide practical tools—such as training, self-assessment, and feedback—may further support implementation for the inpatient and outpatient sector [51,52].

Our study showed awareness of the need for climate action, like previous surveys among German anesthesiologists and outpatient physicians, but suggested that more expert knowledge among staff and support are required to enable the effective implementation of corresponding measures [13,23]. Our study also found a profound lack of knowledge among our sample of healthcare decision-makers regarding pivotal issues, including the recording of greenhouse gas emissions or the consideration of sustainability in product procurement decisions at their facility. These findings align with other studies showing that health professionals primarily associate hospital emissions with energy use and waste, while underestimating the impact of pharmaceuticals and supply chains [45]. National and international initiatives, including non-governmental [7,53,54], governmental [55] and intergovernmental [56] actors, aim to close this knowledge gap by providing guidance and support. Strengthening knowledge transfer and capacity-building could play a key role in more effective climate action at healthcare facilities.

Healthcare decision-makers have a responsibility to shape attitudes within the whole healthcare sector and influence employees, patients, policy makers and society at large [57]. They could significantly accelerate the necessary transformation toward a resilient healthcare system that operates within the planet’s ecological boundaries and can successfully adapt to rapidly changing conditions [24,58]. The potential that might be tapped by as little as a top-down statement of support is illustrated by the fact that nearly 40 % of respondents in the present study were unsure of whether their colleagues and managers support their efforts to mitigate climate change. Many initiatives demonstrate promising progress and provide valuable pathways toward a more sustainable and resilient healthcare sector. However, achieving the necessary scale of change will require more decisive top-down policies, increased funding, and a greater commitment to action. Monitoring key performance indicators is needed to standardize progress, inform resource allocation, and provide stronger evidence for facility-based and policy decisions both within Germany and internationally. Future research should include healthcare workers at the operational level to explore their needs, obstacles and, most importantly, synergies between top-down and bottom-up approaches. Frontline professionals, such as midwives, nurses, and physical therapists, are often the first to identify climate-related health risks. They also play a critical role in mitigation practices through prescribing practices, reduction of unnecessary interventions, and promoting preventive care. Their patient trust and close contact with local populations position them as key drivers of transformative change. Recognizing and integrating their contributions into institutional climate strategies can enhance both the feasibility and effectiveness of mitigation and adaptation measures.

This study had a slight focus on mitigation measures, reflecting the discourse in German health policy at the time [18]. Further, we had to limit the questionnaire’s number of items to ensure appropriate length, which is a reason why adaptation-related items had a focus on exemplary aspects such as infrastructure resilience, heat action plans, and occupational health programs. Future survey iterations could touch on other operational aspects of adaptation included in the WHO’s Operational Framework for Climate-Resilient and Low-Carbon Health Systems, such as service delivery and community engagement [19]. Another limitation of this study is the lack of formal psychometric validation of the survey instrument. While the questionnaire was developed based on established theoretical frameworks and underwent expert review, no validation procedures (e.g., reliability testing, thinking aloud method) were conducted. As such, the findings should be interpreted with appropriate caution, particularly with regard to the internal consistency of the measured constructs. Future research may build upon this work by developing formally validated survey instruments.

The low participation rate matches those of previous surveys, such as Kotcher et al. 2021 (response rate of 2.4 %) [22]. The overall trends of the present study also align with those of national and international studies on health professionals' views on climate change and climate change mitigation [21–23]. Importantly, the sampling frame for the random study population was based on one of the most comprehensive and systematically maintained directory of medical professionals and healthcare facilities in Germany. However, the final sample of respondents cannot be considered representative. For instance, our sample contained a higher proportion of women than the source population (42.2 % vs. 34.4 %). Prior research has shown that women tend to express stronger pro-climate attitudes, perceive higher risks, and support mitigation policies more than men [59]. Greater female leadership has also been linked to stronger sustainability outcomes—for example, companies with over 30 % women on boards adopt more ambitious climate policies, and countries with more women in parliament more often ratify environmental treaties [60]. Our somewhat more female-weighted sample might therefore reflect a leadership cohort more inclined toward climate action, which should be considered when interpreting the findings. Overall, the generalizability of this study is limited by biases common to its design, such as social desirability bias with regards to the items on attitudes, sample biases like self-selection, as well as the low participation rate. One could presume that specialist physicians and healthcare facility management personnel inclined toward and even engaged in climate action would be more likely to participate in the survey, which in turn indicates that overall engagement among healthcare decision-makers in climate change adaptation and mitigation at German healthcare facilities could be lower than implied in our study. Repeated surveys using triangulated sampling techniques would be valuable for capturing a more comprehensive and reliable picture. Nevertheless, this study provides a valuable perspective thanks to its broad, cross-sectoral approach across disciplines and facility types, potentially offering a more inclusive view than prior research, which often focused on specific specialties or professional roles.

## 5 Conclusions

These results indicate that while healthcare decision-makers in Germany recognize the urgency of climate change and express willingness to act, significant gaps remain regarding implementation, structural support, and expert knowledge—particularly regarding allocation of responsible staff, emissions tracking, quality assurance, and adaptation planning. The findings provide a baseline indicating that engagement of German outpatient and inpatient healthcare facilities is growing, despite financial and personnel constraints, and lack of sustainability regulations. Thus, the results underscore the need for stronger top-down policies, dedicated funding mechanisms, and integration of climate-related targets into routine healthcare governance. The low response rate constrains generalizability, yet the broad, cross-sectoral approach offers important insights into how healthcare leadership can enable system-wide transformation. Future interventions should target both institutional leadership and frontline staff, supported by performance metrics to accelerate climate action across German healthcare facilities.

## Funding

This research received no external funding

## Data availability

The datasets generated and/or analyzed during the current study are available from the corresponding author upon reasonable request.

## CRediT authorship contribution statement

**Sophie-Charlotte Sasse:** Writing – review & editing, Writing – original draft, Visualization, Project administration, Methodology, Investigation, Formal analysis, Data curation, Conceptualization. **Frederick Schneider:** Writing – review & editing, Supervision, Methodology, Conceptualization. **Neal Conway:** Writing – review & editing, Methodology, Conceptualization. **Claudia Doblinger:** Writing – review & editing, Supervision, Resources, Conceptualization. **Kai Kolpatzik:** Writing – review & editing, Resources, Investigation, Conceptualization. **Christian M Schulz:** Writing – review & editing, Supervision, Project administration, Methodology, Investigation, Conceptualization. **Adrian A W Baumann:** Writing – review & editing, Writing – original draft, Validation, Supervision, Methodology, Investigation, Data curation, Conceptualization. **Nikolaus Christian Simon Mezger:** Writing – review & editing, Visualization, Validation, Supervision, Project administration, Methodology, Investigation, Formal analysis, Data curation.

## Declaration of competing interest

The authors declare no conflicts of interest. CMS and NCSM are members of the German Alliance on Climate Change and Health. NCSM is a member of the steering committee of the Planetary Health Alliance.

## Appendix Supplementary materials

Image, application 1

Image, application 2
